# Tongxinluo-pretreated mesenchymal stem cells facilitate cardiac repair via exosomal transfer of *miR-146a-5p targeting* IRAK1/NF-κB p65 pathway

**DOI:** 10.1186/s13287-022-02969-y

**Published:** 2022-07-07

**Authors:** Yuyan Xiong, Ruijie Tang, Junyan Xu, Wenyang Jiang, Zhaoting Gong, Lili Zhang, Yu Ning, Peisen Huang, Jun Xu, Guihao Chen, Xiaosong Li, Mengjin Hu, Jing Xu, Chunxiao Wu, Chen Jin, Xiangdong Li, Haiyan Qian, Yuejin Yang

**Affiliations:** State Key Laboratory of Cardiovascular Disease, Department of Cardiology, Fuwai Hospital, National Center for Cardiovascular Diseases, Chinese Academy of Medical Science and Peking Union Medical College, Beijing, 10037 China

**Keywords:** Myocardial infarction, Mesenchymal stem cells, Exosomes, Tongxinluo, Pretreatment

## Abstract

**Background:**

Bone marrow cells (BMCs), especially mesenchymal stem cells (MSCs), have shown attractive application prospects in acute myocardial infarction (AMI). However, the weak efficacy becomes their main limitation in clinical translation. Based on the anti-inflammation and anti-apoptosis effects of a Chinese medicine-Tongxinluo (TXL), we aimed to explore the effects of TXL-pretreated MSCs (MSCs^TXL^) in enhancing cardiac repair and further investigated the underlying mechanism.

**Methods:**

MSCs^TXL^ or MSCs and the derived exosomes (MSCs^TXL^-exo or MSCs-exo) were collected and injected into the infarct zone of rat hearts. In vivo, the anti-apoptotic and anti-inflammation effects, and cardiac functional and histological recovery were evaluated. In vitro, the apoptosis was evaluated by western blotting and flow cytometry. miRNA sequencing was utilized to identify the significant differentially expressed miRNAs between MSCs^TXL^-exo and MSCs-exo, and the miRNA mimics and inhibitors were applied to explore the specific mechanism.

**Results:**

Compared to MSCs, MSCs^TXL^ enhanced cardiac repair with reduced cardiomyocytes apoptosis and inflammation at the early stage of AMI and significantly improved left ventricular ejection fraction (LVEF) with reduced infarct size in an exosome-dependent way. Similarly, MSCs^TXL^-exo exerted superior therapeutic effects in anti-apoptosis and anti-inflammation, as well as improving LVEF and reducing infarct size compared to MSCs-exo. Further exosomal miRNA analysis demonstrated that *miR-146a-5p* was the candidate effector of the superior effects of MSCs^TXL^-exo. Besides, *miR-146a-5p* targeted and decreased IRAK1, which inhibited the nuclear translocation of NF-κB p65 thus protecting H9C2 cells from hypoxia injury.

**Conclusions:**

This study suggested that MSCs^TXL^ markedly facilitated cardiac repair via a new mechanism of the exosomal transfer of *miR-146a-5p* targeting IRAK1/NF-κB p65 pathway, which has great potential for clinical translation.

**Supplementary Information:**

The online version contains supplementary material available at 10.1186/s13287-022-02969-y.

## Background

Ischemic heart disease has long been a major cause of cardiovascular disease mortality worldwide despite improved medical care and even reperfusion therapy for acute myocardial infarction (AMI) [1, 2]. Sudden loss of massive cardiomyocytes accompanied by an intense inflammatory response in the early stage of AMI correlated directly with deteriorated cardiac function, ventricular remodeling, heart failure and worse clinical outcomes [3–5]. Bone marrow cell (BMC) transplantation, including mesenchymal stem cell (MSC) transplantation, remains a promising method for the treatment of patients with AMI and ischemic heart failure [6]. However, the weak therapeutic effects of transplanted BMCs and MSCs have restricted their clinical applications [7–11]. Although numerous studies have explored different strategies to enhance the effects of MSCs including preconditioning [12, 13], genetic modification [14, 15] and tissue engineering [16, 17], some of these strategies are still challenging with respect to clinical translation in current AMI treatment.

Our group has explored a clinically feasible strategy to enhance the therapeutic efficacy of MSCs by pretreatment with the widely used clinical medicine statins and the Chinese medicine compound Tongxinluo (TXL) in preclinical and clinical studies [18–21]. Both statins and TXL share similar anti-inflammatory and anti-apoptotic mechanisms, resulting in microenvironment improvement in the post-infarct myocardium and facilitating the survival of implanted MSCs [18–20]. Statins and TXL have also been confirmed to protect MSCs from hypoxia and serum deprivation (H/SD)-induced apoptosis in vitro [22, 23] and exerted anti-apoptotic effects in vivo [24, 25]. In addition, MSCs pretreated with statins demonstrated marked therapeutic potential [26–28], though the effects of TXL-pretreated MSCs (MSCs^TXL^) have not yet been explored. We herein aimed to explore whether MSCs^TXL^ could enhance cardiac repair effects in AMI and the underlying mechanism.

The therapeutic effects of MSCs on cardiac repair have been attributed to their paracrine mechanism [29] including microvesicles and exosomes, which play pivotal roles in intercellular communication [30, 31]. Among these, MSCs-derived exosomes exhibit distinct benefits in improving harsh environments and promoting cardiac recovery after AMI [32]. Exosomes, 30–150 nm extracellular vesicles, manifest several favorable features including low immunogenicity and reduced biodegradability and are capable of carrying a complex cargo of bioactive molecules including proteins, lipids and RNAs to mediate intercellular communications [33, 34]. MicroRNAs (miRNAs) are a class of short noncoding RNAs regulating gene expression at the posttranslational level [35], and exosomal miRNAs have been found to play an important role in cardiac repair [36]. Therefore, we hypothesized that miRNAs in MSCs^TXL^-derived exosomes (MSCs^TXL^-exo) might be essential to the MSCs^TXL^-mediated cardioprotective effects in AMI.

In the present study, we first reported the superior efficacy of MSCs^TXL^ relative to MSCs especially in anti-apoptosis and anti-inflammation at an early stage, as well as cardiac function recovery in an exosome-dependent manner. Further analysis found that exosomal *miR-146a-5p* levels were upregulated and then transferred to cardiomyocytes, which targeted the IRAK1/NF-κB p65 signaling pathway, thus ameliorating cardiomyocyte injury. These findings provide new insights into the novel mechanism of MSCs^TXL^ in enhancing the effects of cardiac repair in AMI.

## Methods

All animals were obtained from the Animal Department of Fuwai Hospital. The experiment was performed with approval of the Experimental Animals Ethics Committee of Fuwai Hospital (FW-2018-0010), and all procedures conformed to National Institutes of Health guidelines. A more detailed description of the experimental procedures is available in the Additional file 1.

### MSCs isolation and pretreatment

Sprague–Dawley (SD) rats were housed in a pathogen-free conditions under a controlled 12-h light–dark cycle and allowed free access to water and chow. Bone marrow was flushed with medium from the tibia and femur of male SD rats (60–80 g) and cultured in complete medium (IMDM, 10% fetal bovine serum) in 5% CO_2_ at 37 °C. After 24 h, remove the nonadherent cells that accumulate on the surface of the dish by changing the medium and replacing with fresh IMDM with 10% FBS and penicillin (100 U/mL)/streptomycin (100 μg/mL). MSCs at passages 3–4 appeared spindle shaped and were identified by canonical surface marker probing as CD45^−^, CD11^−^, CD31^−^, CD29^+^, CD90^+^, CD73^+^. Passage 3–4 MSCs were pretreated with TXL solution for 24 h.

### Exosome extraction, identification and labeling

Exosomes were isolated by differential centrifugation. Briefly, MSCs were cultured in exosome-free FBS containing IMDM for 48 h. The conditioned supernatants of MSCs pretreated with TXL or not were then collected and centrifuged. The conditioned supernatants were first centrifuged at 300 g for 10 min and 2000 g for 20 min to remove cells and debris and then centrifuged at 16500 g for 30 min to eliminated large extracellular vesicles. The crude exosome pellet was obtained by ultracentrifuging at 120000 g for 70 min, then the pellet was washed in Phosphate-buffered saline (PBS, pH7.4) followed by repeat ultracentrifugation for 70 min at the same speed. The exosome pellet was then suspended in appropriate volume of PBS and stored at −80 °C for the use in the experiments.

Protein concentrations of exosome were measured by microBCA protein assay kit (Thermo Fisher Scientific). Shape and sizes of exosomes were identified by transmission electron microscope (TEM, FEI, Tecnai G2 Spirit BioTwin) and Nanoparticle Tracking Analysis (NTA, PARTICLE METRIX, ZetaVIEW). Additionally, exosomes were identified by using western blotting with antibodies against CD63, TSG101, and Alix, whose catalog numbers are listed in Additional file 1: Table S1.

To determine whether exosomes can be effectively taken, purified exosomes were labeled with fluorescent dye PKH26 using Red Fluorescent Cell Linker Kit (Sigma-Aldrich) according to the manufacturer’s instructions and washed in PBS followed by two times of ultracentrifugation to remove the extra dye. After 12, or 24 h incubation with pre-labeled exosomes, H9C2 cells were then washed with PBS, fixed with 4% paraformaldehyde, then stained with phalloidine and DAPI at room temperature. For in vivo experiments, PKH26 pre-labeled exosomes were injected into the border zone of the infarcted heart and the distribution of pre-labeled exosomes were then monitored by confocal microscopy.

### AMI model establishment

Animals were randomized into different groups. Female SD rats (200–220 g weight) were anesthetized by intraperitoneal injection of pentobarbital sodium (50 mg/kg) before the surgical procedure. A 6–0 polyester suture was used to ligate the left anterior descending coronary artery (LAD). Thirty minutes after ligation, MSCs (2 × 10^6^ MSCs, in 100 μL PBS) or exosomes (20 μg, in 100 μL PBS) or 100 μL PBS was injected into the border zone of the infarcted heart at three sites. Transthoracic two-dimensional M-mode echocardiography was performed at 3 days (baseline) and 28 days (endpoint) post-AMI with VisualSonics Vevo 2100 system after rats were anesthetized with isoflurane. Rats were then placed on a heating pad, keeping the heart rate > 350 b.p.m. and the core body temperature ~ 37 °C. Left ventricular ejection fraction (LVEF), left ventricular fractional shortening (LVFS), left ventricular end-diastolic volume (LVEDV), and left ventricular end-systolic volume (LVESV) were assessed and calculated as previously described [37].

### Histological analysis

Rats were killed after echocardiography measurements were recorded. Masson’s trichrome and Sirius red staining were used to quantify the extent of the infarct size and fibrosis in the left ventricle (LV) with ImageJ software. Infarct size was calculated as [(epicardial infarct ratio + endocardial infarct ratio)/2] × 100. The epicardial infarct ratio was obtained by dividing epicardial infarct length by the epicardial circumference. The endocardial infarct ratio was calculated similarly. Collagen area was quantified as the average ratio of collagen area to the total LV area (collagen area/total LV area × 100%). Hematoxylin–eosin (HE) staining was used to roughly evaluate the degree of inflammatory cell infiltration. Vascular density was quantified by immunofluorescence of α-SMA and CD31 as described in the Additional file 1. The number of apoptotic cardiomyocytes was counted by terminal deoxynucleotidyl transferase-mediated dUTP nick-end labeling (TUNEL) assay, as described in the Additional file 1.

### Apoptosis determination by flow cytometry

H9C2 cells, pretreated by exosomes for 24 h or transfected with miRNA mimics, inhibitors or siRNA, were exposed to H/SD condition. Then H9C2 cells were collected and measured by using the Annexin V-FITC/PI Kit (Becton, Dickinson and Company) according to the manufacturer’s protocol. Viable cells were defined as Annexin V^−^/PI^−^, early apoptotic cells as Annexin V^+^/PI^−^, late apoptotic and necrotic cells as annexin V^+^/PI^+^. The proportion of apoptotic cells was calculated after adding together early and late apoptotic cells.

### miRNA mimics or inhibitors and siRNA transfection

*miR-146a-5p* mimics, inhibitors, interleukin 1 receptor-associated kinase 1 (*Irak1*) siRNA and their negative controls (NCs) were purchased from RiboBio. Target cells (MSCs or H9C2 cells) were transfected with *miR-146a-5p* mimics (100 nM), or *miR-146a-5p* inhibitors (200 nM), *Irak1* siRNA (100 nM) or relative NC (100–200 nM) by using Lipofectamine RNAiMAX (Invitrogen) according to manufacturer’s instructions. After 6 h of incubation, the medium was replaced with fresh medium, and then the cells were cultured for 48 h.

### Exosomal miRNA sequencing

The miRNA sequencing was performed both in MSCs-derived exosomes (MSCs-exo) and MSCs^TXL^-derived exosomes (MSCs^TXL^-exo). Three samples were processed for each group. Exosomal RNA was used to prepare miRNA next-generation sequencing (NGS) libraries with the QIAseq miRNA Library Kit. The libraries obtained from different samples were then performed on an Illumina Hiseq X Ten platform. Differentially expressed miRNAs were identified by |fold change| > 2 and *p* value < 0.05 with the threshold set for up- and downregulated genes.

### Dual-luciferase reporting system

HEK293T cells were plated in a 96-well plate at 1 × 10^4^ cells/well for 24 h, and transfection was performed once the cells achieved 60–70% confluency. Lipo6000™ (Beyotime Biotechnology) was used to transfect the cells. After 48 h transfection, miRNA level was detected by chemiluminescence using the Dual-Luciferase Reporter Assay System (Promega).

### Quantitative real-time-polymerase chain reaction (qRT-PCR)

Total RNA and miRNA were extracted from exosomes, cells and tissues with Trizol reagent (Life Technologies) according to the manufacturer’s instructions. mRNAs reverse transcription was performed using a Prime-Script^TM^RT Reagent Kit with gDNA Eraser (Takara) and miRNAs were revere transcribed with a Bulge-Loop miRNA qRT-PCR Starter Kit (RiboBio). The quantitative real-time PCR process was conducted with PowerUp™ SYBR™ Green Master Mix (Applied Biosystem) on a QuantStudio 3 Real-Time PCR system (Applied Biosystem). The level of mRNA and miRNA was normalized to GAPDH and U6, respectively, and the data were calculated via comparative 2^−ΔΔCt^ methods. Bulge-Loop miRNA qRT-PCR primer sequence was designed and synthesized by RiboBio, and the mRNA primer sequences were the synthesized by TianyBiotech and listed in Additional file 1: Table S2. Each experiment included three technical replicates and at least three independent repeats.

### Statistical analysis

All data are expressed as the mean ± standard deviation (SD). Statistical analyses were performed using GraphPad 8.0 (GraphPad Software, USA). Comparisons between two groups were compared by Student’s t test, and comparisons among three or more groups were evaluated by one-way ANOVA followed by Tukey’s test. The level of significance was set at *p* < 0.05 for all comparisons.

## Results

### MSCs^TXL^ improved heart function and reduced cardiomyocyte apoptosis and inflammation after AMI

**Fig. 1:**
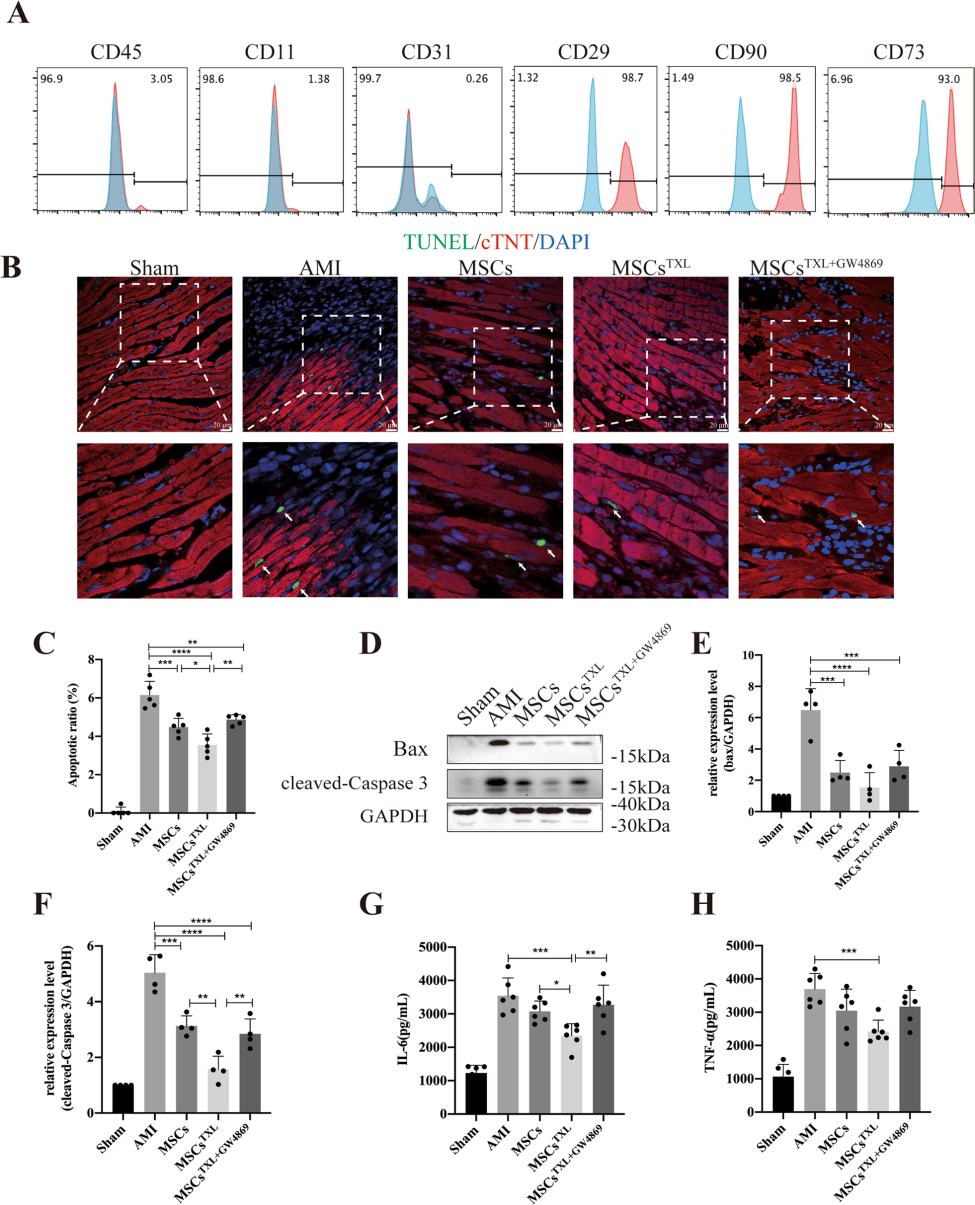
Intramyocardial injection of MSCs^TXL^ significantly ameliorated cardiomyocyte apoptosis and limited inflammation at the early stage of AMI. **A** Flow cytometry indicated that MSCs were negative for CD45, CD11, CD31 and positive for CD90, CD29 and CD73. **B** Representative images of TUNEL staining 3 days after AMI. The regions boxed with white dashed lines are enlarged in the lower panel. Cardiomyocytes were stained with cTNT (red) and nuclei were stained blue. The arrows show the apoptotic cardiomyocytes. Scale bar = 20 μm. **C** Quantification of cTNT^+^TUNEL^+^ cardiomyocytes (4 random fields per animal; *n* = 5). Representative western blotting images (**D**) of Bax, cleaved-Caspase 3 and GAPDH and the relative level of Bax (**E**) and cleaved-Caspase 3 (F) (*n* = 4). GAPDH was used as a loading control. **G** and **H** Quantification of IL-6 and TNF-α levels in infarct border zone tissue of rat hearts at 3 days post-AMI using ELISA (*n* = 6). All data are expressed as the mean ± SD. Statistical analysis was performed with one-way ANOVA followed by Tukey’s test. *p < 0.05, **p < 0.01, ***p < 0.001, ****p < 0.0001

Bone marrow-derived MSCs at passages 3–4 appeared spindle shaped and were further identified by canonical surface marker probing as CD45^−^, CD11^−^, CD31^−^, CD29^+^, CD90^+^, CD73^+^ (Fig. 1A). MSCs could differentiate into adipocytes, osteocytes and chondrocytes, as confirmed by oil red O staining, alizarin red staining and alcian blue staining, respectively (Additional file 1: Fig. S1A). To assess the appropriate pretreatment concentrations of TXL, different concentrations of TXL were applied to MSCs. The cell viability of MSCs obviously decreased under normal conditions when the concentration was above 800 μg/mL, while a 100–400 μg/mL concentration resulted in a dose-dependent increase in cell viability under H/SD conditions, as shown by the CCK-8 assay (Additional file 1: Fig. S1B). Thus, we chose a concentration of 400 μg/mL for the pretreatment of MSCs (MSCs^TXL^). Further analysis showed that MSCs^TXL^ had similar surface markers and differentiation ability as MSCs (Additional file 1: Fig. S1C-D).

To investigate whether TXL pretreatment could enhance the therapeutic efficiency of MSCs, MSCs pretreated with TXL (MSCs^TXL^ group), MSCs (MSCs group), or PBS (AMI group) was intramyocardially injected into the border zone of infarcted rat hearts 30 min post-AMI. Three days post injection, the MSCs^TXL^ group revealed fewer apoptotic cardiomyocytes (cTNT^+^TUNEL^+^) in the ischemic border zone than those receiving PBS or MSCs treatment (Fig. 1B, C). Western blot analysis also indicated reduced apoptosis in the MSCs^TXL^ group as the pro-apoptotic Bax and cleaved-Caspase 3 level was decreased significantly in the MSCs^TXL^ group (Fig. 1D–F, Additional file 1: Table S3). Furthermore, HE staining roughly showed decreased infiltration of inflammatory cells in the MSCs^TXL^ group (Additional file 1: Fig. S2A), and the ELISA results indicated the decreased levels of inflammatory cytokines including IL-6 and TNF-α, in the infarct border zone (Fig. 1G, H, Additional file 1: Table S3).

**Fig. 2:**
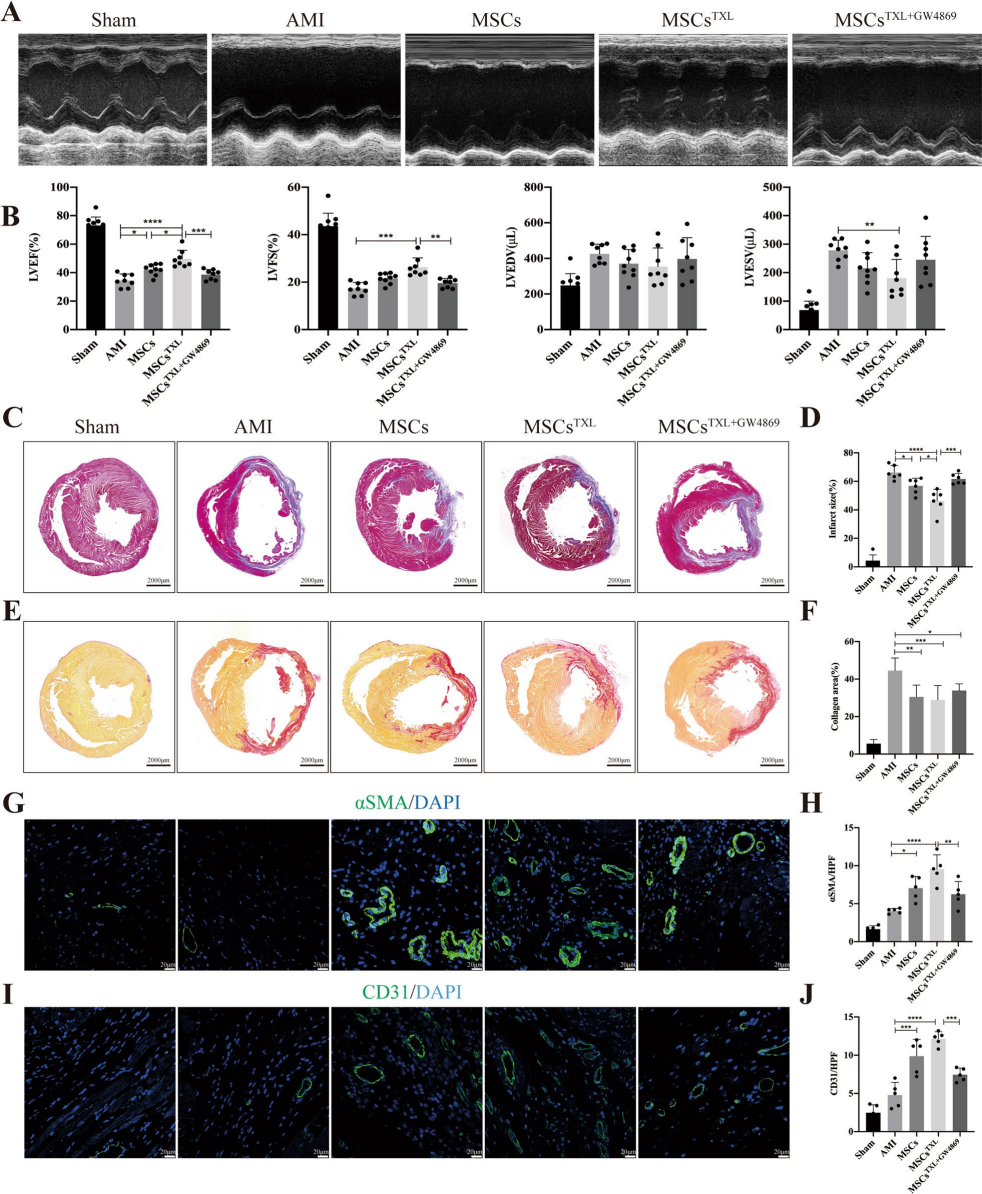
Intramyocardial injection of MSCs^TXL^ improved cardiac function, reduced infarct size and promoted angiogenesis after AMI. Representative images (**A**) of echocardiograms of rat heart and quantitative analysis (**B**) of LVEF, LVFS, LVEDV and LVESV (*n* = 8–10 for each group) at 28 days post-AMI. Representative images (**C**) and quantification (**D**) of the LV infarct size (*n* = 6) of transverse heart sections with Masson’s trichrome staining at 4 weeks after AMI. Scale bar = 2000 μm. Representative images of Sirius red staining (**E**) and quantification (**F**) of the collagen area. Scale bar = 2000 μm. Representative images of αSMA positively stained arterioles (**G**) or CD31 positively stained capillaries (**I**) at the border zone. Scale bar = 20 μm. Quantification of arteriole density (**H**) or capillary density (**J**) of the corresponding images of (**G**) or (**I**) (*n* = 5). Data were analyzed with one-way ANOVA followed by Tukey’s test and are shown as the mean ± SD. *p < 0.05, **p < 0.01, ***p < 0.001, ****p < 0.0001

Four weeks post-AMI, both MSCs and MSCs^TXL^ administration exhibited significant improvement in LVEF compared to the AMI group, while MSCs^TXL^ demonstrated a therapeutic superiority to MSCs in systolic function improvement (49.69 ± 6.02% vs. 42.10 ± 3.83%, *p* < 0.05) (Fig. 2A, B, Additional file 1: Table S3), with a significantly smaller infarct size in the MSCs^TXL^ group compared to the MSCs (46.39 ± 8.06% vs. 56.82 ± 5.41%, *p* < 0.05) and AMI groups (46.39 ± 8.06% vs. 66.29 ± 4.96%, *p* < 0.0001) by Masson’s trichrome staining analysis (Fig. 2C, D, Additional file 1: Table S3). In addition, the collagen area determined by Sirius red staining decreased significantly in both the MSCs^TXL^ and MSCs group (*p* < 0.01–0.001), while there was no obvious difference between them (Fig. 2E, F). Arteriole density (determined by α-SMA immunofluorescence staining) and capillary density (determined by CD31 immunofluorescence staining) in the ischemic border zone were both significantly increased in the MSCs^TXL^ and MSCs group (*p* < 0.05–0.0001) (Fig. 2G–J, Additional file 1: Table S3).

Taken together, these data indicated that TXL pretreatment enhanced the therapeutic efficacy of MSCs in cardiac repair, especially in protecting cardiomyocytes against apoptosis and limiting inflammation at an early stage of AMI.

### GW4869 abolished the superior effects of MSCs^TXL^

As a blocker of neutral sphingomyelinase that inhibits exosome secretion, GW4869 could inhibit exosome production in a dose-dependent manner with complete blockage at a concentration of 20 μM which do not affect cell viability [38–40]. After pretreatment of MSCs^TXL^ with 20 μM GW4869 for 24 h, we found that GW4869 pretreatment effectively blocked the release of exosomes (Additional file 1: Fig. S2B) and abrogated the effects of MSCs^TXL^ in reducing apoptosis, limiting inflammation (Fig. 1B–H, Additional file 1: Table S3), improving cardiac function, decreasing infarct and fibrotic size, and promoting angiogenesis (Fig. 2A–J, Additional file 1: Table S3). Therefore, these results indicated that exosomes derived from MSCs^TXL^ are vital for cardiac repair.

### MSCs^TXL^-exo exhibited better effects in inhibiting cardiomyocyte apoptosis in vitro

**Fig. 3:**
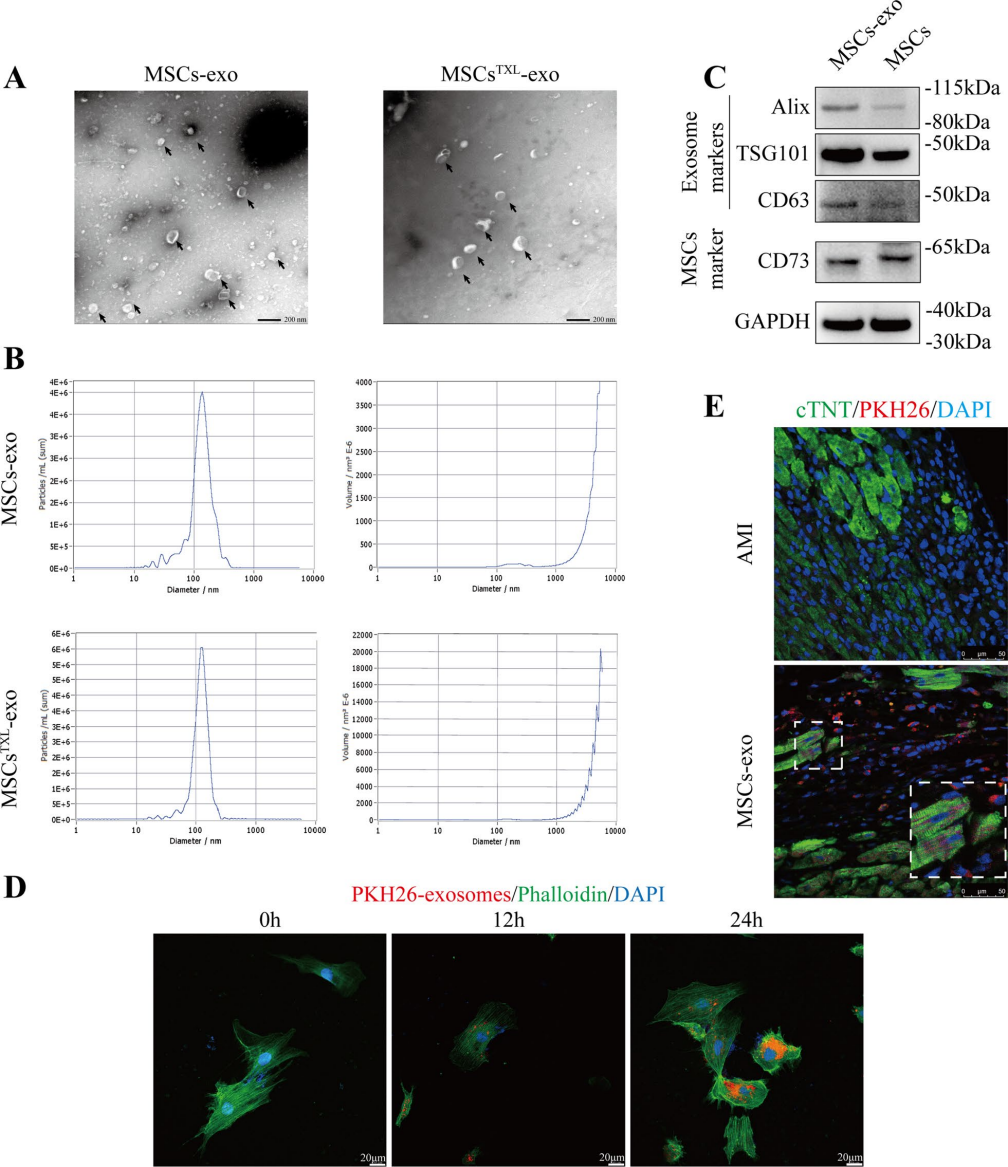
Characterization of exosomes derived from MSCs. **A** MSCs-exo and MSCs^TXL^-exo were assessed by TEM and exhibited cup-shaped morphology. Scale bar = 200 μm. **B** NTA was performed to characterize the particle size and concentration. **C** Representative western blotting images of exosomal protein markers and MSCs markers. **D** Representative confocal images indicated that PKH26 labeled exosomes were taken up by H9C2 cells after 12 or 24 h of incubation. Scale bar = 20 μm. **E** PKH26-labeled MSCs-exo could be taken up by cardiomyocytes in infarcted hearts on Day 3 post-AMI. Scale bar = 50 μm

Since the cardioprotective roles of MSCs against MI injury were mainly mediated by exosomes [39, 41, 42] and those of MSCs^TXL^ were also abolished by GW4869 pretreatment, we further investigated whether MSCs^TXL^ facilitate cardiac repair via exosomes. Exosomes, isolated by ultracentrifugation, were characterized by TEM (Fig. 3A) as having similar particle sizes and concentrations between MSCs-derived exosomes (MSCs-exo) and MSCs^TXL^-exo (Fig. 3B). The exosomes were positive for exosome-specific markers including Alix, Tsg101 and CD63, as well as the MSCs surface marker CD73 (Fig. 3C). H9C2 cells were cocultured with PKH26-labeled exosomes for 12 and 24 h, and exosomal uptake was further verified via PKH26 fluorescence images. As shown in Fig. 3D, the uptake rate was positively correlated with the cocultivation time. Three days after PKH26-labeled exosome injection, confocal images indicated that these injected exosomes could be taken up by cardiomyocytes in vivo (Fig. 3E).

**Fig. 4:**
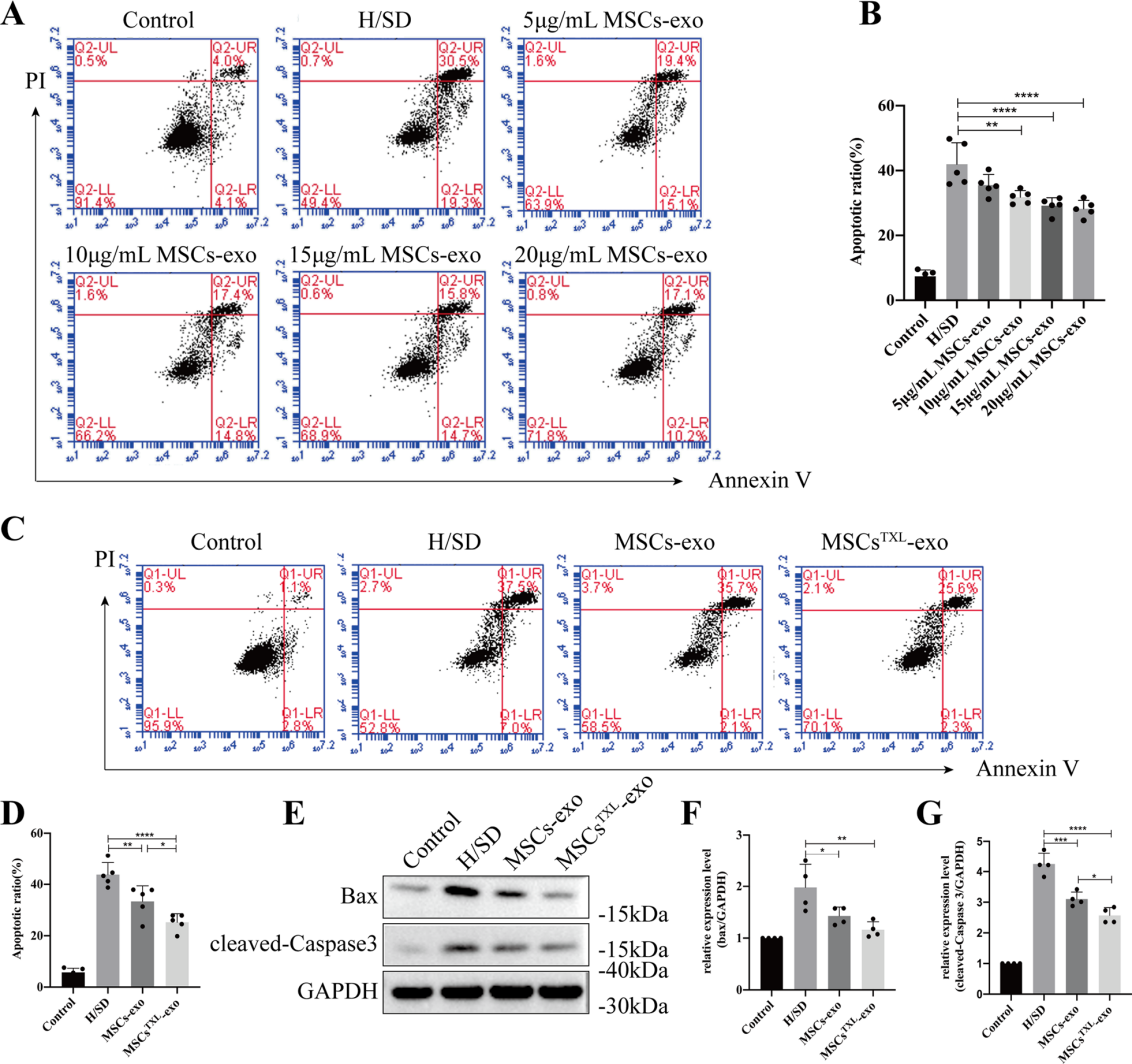
Exosomes derived from MSCs^TXL^ protected H9C2 cells against apoptosis. Representative images of the flow cytometry assay (**A**) and quantification (**B**) of apoptosis of H9C2 cells after pretreatment with different concentrations of MSCs-exo (*n* = 5). Representative images of flow cytometry (**C**) and quantification (**D**) of apoptosis of H9C2 cells pretreated with 10 μg/mL MSCs-exo or MSCs^TXL^-exo under H/SD conditions (*n* = 5). Representative images of western blotting (**E**) and quantification of Bax (**F**) and cleaved-Caspase 3 (**G**) levels after exosome pretreatment (*n* = 4). Data were analyzed with one-way ANOVA followed by Tukey’s test and are presented as the mean ± SD. *p < 0.05, **p < 0.01, ***p < 0.001, ****p < 0.0001

In vitro, to evaluate the protective effects of exosomes, H9C2 cells were pretreated with different concentrations of MSCs-exo for 24 h and then subjected to H/SD. Treatment with exosomes at a concentration of 10–20 μg/mL resulted in a dose-dependent decrease in apoptosis compared to H/SD group, with no significant difference among the 10, 15 and 20 μg/mL groups (Fig. 4A, B). Thus, we chose a concentration of 10 μg/mL for the following in vitro assays. When the same concentration (10 μg/mL) of MSCs-exo and MSCs^TXL^-exo was applied to H9C2 cells, the apoptotic ratio significantly dropped in both the MSCs-exo and MSCs^TXL^-exo treated group compared to the H/SD group (33.34 ± 6.07% and 25.22 ± 3.35% vs. 43.78 ± 4.70%, *p* < 0.01 and *p* < 0.0001, respectively), with a significantly more decrease in the latter group than the former one (*p* < 0.05) (Fig. 4C, D, Additional file 1: Table S4). Further western blot analysis validated that MSCs^TXL^-exo were superior to MSCs-exo in decreasing the level of cleaved-Caspase 3 (Fig. 4E–G, Additional file 1: Table S4). These results demonstrated the superior protective effects of MSCs^TXL^-exo compared to MSCs-exo in protecting H9C2 cells against hypoxia-induced apoptosis.

### MSCs^TXL^-exo demonstrated better functional recovery by reducing apoptosis and suppressing inflammation

**Fig. 5:**
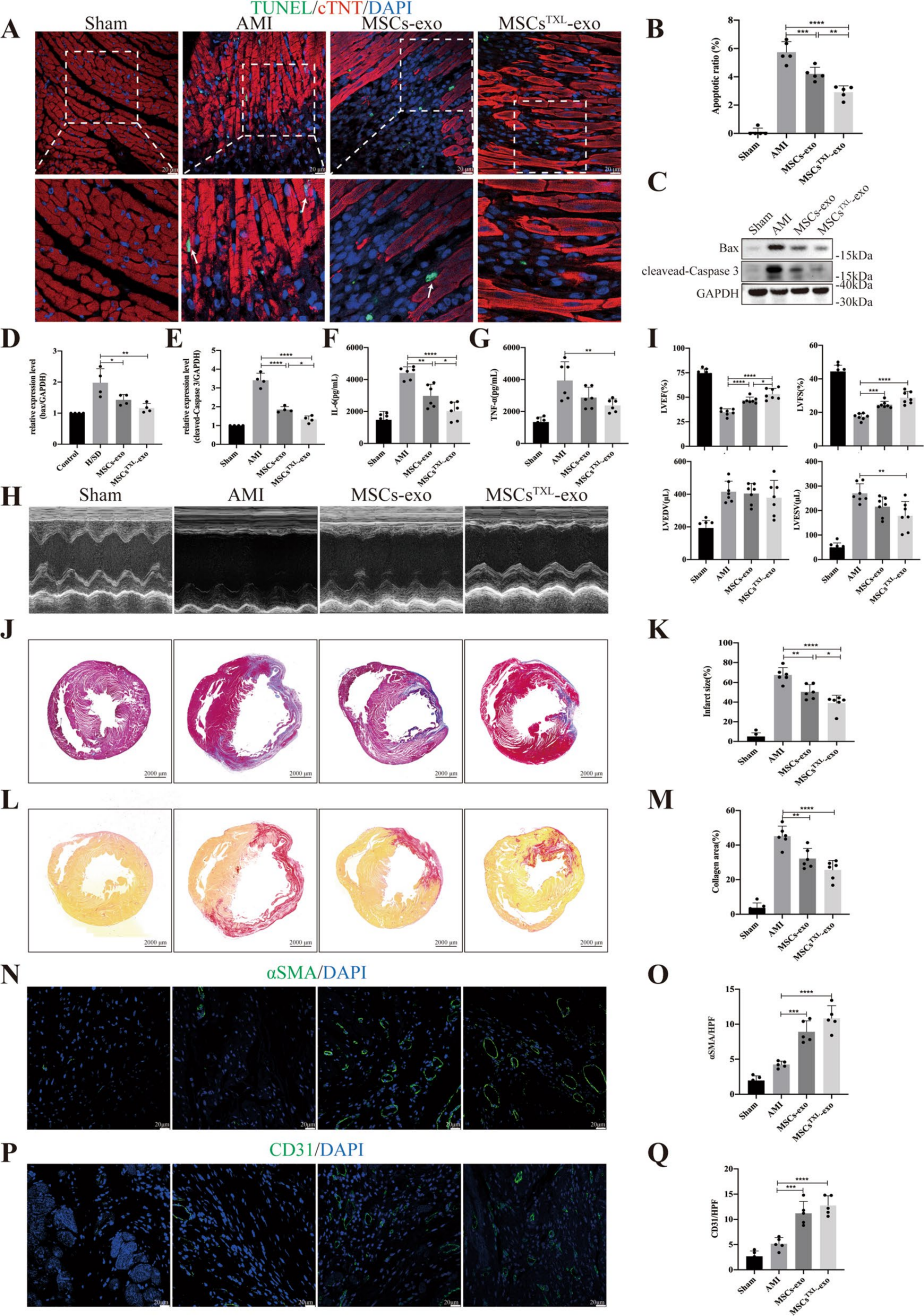
MSCs^TXL^-exo exhibited better therapeutic efficacy in decreasing apoptotic cardiomyocytes, limiting inflammation, improving cardiac function, reducing infarct size, and cardiac fibrosis and promoting angiogenesis. Representative TUNEL staining images (**A**) and quantification of cTNT^+^TUNEL^+^ cardiomyocytes (**B**) (4 random fields per animal; *n* = 5) at 3 days post AMI. The regions boxed with white dashed lines are enlarged in the lower panel. The cardiomyocytes were stained with cTNT (red), and nuclei were stained blue. Scale bar = 20 μm. (**C**) Representative western blotting images of Bax, cleaved-Caspase 3 and GAPDH, and the Bax (**D**) and cleaved-Caspase 3 level (**E**) (*n* = 4). (**F**) and (**G**) Quantification of IL-6 and TNF-α levels in the infarct border zone at 3 days post-AMI (*n* = 6). Representative images of echocardiograms (**H**) and quantitative analysis (**I**) of LVEF, LVFS, LVEDV, and LVESV at 28 days post-AMI (*n* = 7–8 for each group). Representative images (**J**) and quantitative analysis (**K**) of Masson’s trichrome staining at 4 weeks post-AMI. Scale bar = 2000 μm (*n* = 6). Representative images of Sirius red staining (**L**) and quantification (**M**) for collagen area. Scale bar = 2000 μm (*n* = 6). Representative images of αSMA positively stained arterioles (**N**) or CD31 positively stained capillaries (**P**) at the border zone and the corresponding quantitative data (**O**) or (**Q**) (*n* = 5). Scale bar = 20 μm. Data were analyzed with one-way ANOVA followed by Tukey’s test and are shown as the mean ± SD. *p < 0.05, **p < 0.01, ***p < 0.001, ****p < 0.0001

We further compared the therapeutic efficacy between MSCs^TXL^-exo and MSCs-exo in vivo. Consistent with previous results of animals receiving MSCs and MSCs^TXL^, the MSCs^TXL^-exo group exhibited significantly fewer apoptotic cardiomyocytes (cTNT^+^TUNEL^+^), lower levels of pro-apoptotic Bax and cleaved-Caspase 3 and inflammatory cytokines, as well as a higher LVEF and smaller infarct size (Fig. 5A–K, Additional file 1: Table S5) when compared to the MSCs-exo group. The MSCs^TXL^-exo group also showed a trend of collagen area reduction and angiogenesis promotion over the MSCs-exo group, although the difference was not significant (Fig. 5L–Q, Additional file 1: Table S5). These results indicated that MSCs^TXL^-exo had superior cardioprotective effects than MSCs-exo in vivo, especially in reducing apoptosis and suppressing inflammation.

### ***miR-146a-5p*** was the candidate effector of MSCs^TXL^-exo in mediating cardioprotective effects

**Fig. 6:**
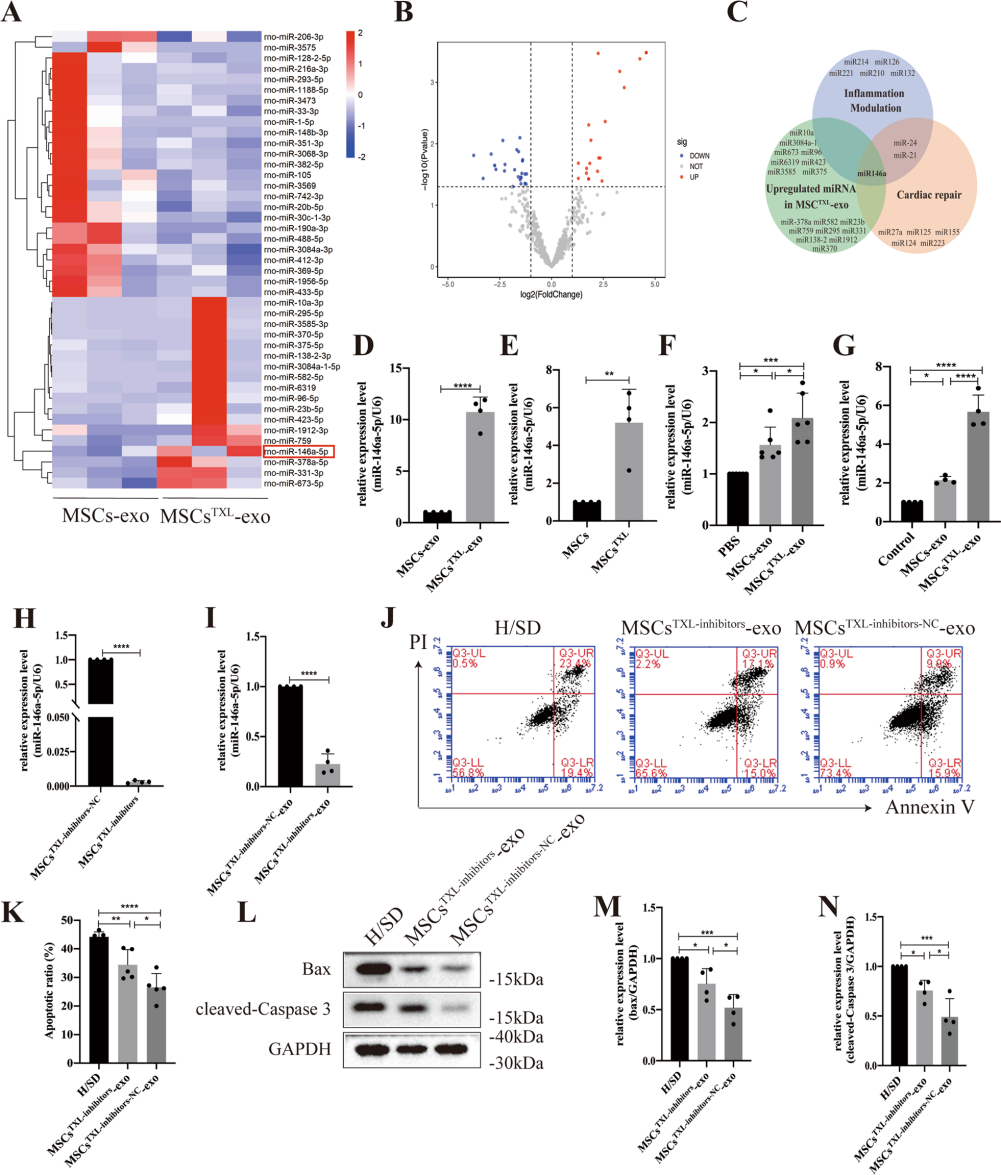
Significant differences in the miRNA expression profile between MSCs-exo and MSCs^TXL^-exo. **A** Heatmap of upregulated and downregulated miRNAs in MSCs^TXL^-exo compared to MSCs-exo (*n* = 3). **B** Volcano plot showing the significantly differentially expressed miRNAs (twofold change and *p* < 0.05 as the threshold) in MSCs^TXL^-exo compared to MSCs-exo. **C** The overlap of upregulated miRNAs in MSCs^TXL^-exo according to miRNA sequencing analysis and the main reported miRNAs participating in inflammation modulation and cardiac repair. *miR-146a-5p* stood out as the candidate effector. qRT-PCR validation of *miR-146a-5p* levels in exosomes (**D**) (*n* = 4) and MSCs (E) (*n* = 4). **F** *miR-146a-5p* levels in the infarct border zone of PBS, MSCs-exo or MSCs^TXL^-exo-treated rat hearts (*n* = 6). **G** *miR-146a-5p* levels in H9C2 cells that were cocultured with PBS, MSCs-exo or MSCs^TXL^-exo for 24 h (*n* = 4). *miR-146a-5p* levels in MSCs^TXL^ (**H**) or exosomes (**I**) derived from MSCs^TXL^ treated with *miR-146a-5p* inhibitors or its negative control (NC) (*n* = 4). **J** Representative images of the flow cytometry assay and quantification (**K**) of apoptosis after treatment with MSCs^TXL-inhibitors^-exo or MSCs^TXL-inhibitors-NC^-exo (*n* = 5). Western blotting images (**L**) and quantification of Bax (**M**) and cleaved-Caspase 3 (**N**) levels in H9C2 cells after treatment with MSCs^TXL-inhibitors^-exo or MSCs^TXL-inhibitors-NC^-exo (*n* = 4). All data are expressed as the mean ± SD and were analyzed with Student’s t test or one-way ANOVA. *p < 0.05, **p < 0.01, ***p < 0.001, ****p < 0.0001

Since exosomal miRNAs are vital in intercellular communication, we sought to investigate the miRNA expression profiles between MSCs^TXL^-exo and MSCs-exo to further elucidate the mechanism. miRNA sequencing analysis demonstrated 43 differentially expressed miRNAs, with a twofold change and *p* < 0.05 threshold cutoff (Fig. 6A, B). Among the differentially expressed miRNAs, 18 miRNAs were upregulated in MSCs^TXL^-exo. Based on the superior effects of MSCs^TXL^-exo in inflammation modulation and cardiac repair, we searched for widely reported miRNAs involved in either inflammation modulation (*miR-214*, *miR-126*, *miR-221*, *miR-210*, *miR-132*, *miR-146a*, *miR-24* and *miR-21*) or cardiac repair (*miR-27a*, *miR-125*, *miR-155*, *miR-124*, *miR-223*, *miR-146a*, *miR-21* and *miR-24*). *miR-146a* stood out as a candidate effector, which is enriched in MSCs^TXL^-exo and probably responsible for reducing apoptosis and suppressing inflammation after AMI, as shown in Fig. 6C. To validate the miRNA-profile results, we tested the levels of *miR-146a-5p* in MSCs-exo and MSCs^TXL^-exo. Compared to MSCs-exo, TXL pretreatment significantly upregulated the level of *miR-146a-5p* in MSCs^TXL^-exo (> tenfold, *p* < 0.0001) (Fig. 6D). In addition, TXL pretreatment also increased *miR-146a-5p* levels in MSCs^TXL^ compared to MSCs (*p* < 0.01) (Fig. 6E).

To explore the relationship between *miR-146a-5p* and the cardioprotective effects of TXL pretreatment, we measured the *miR-146a-5p* levels in the infarct border zone of rat hearts 4 weeks post-AMI. qPCR analysis confirmed the significantly higher level of *miR-146a-5p* in the MSCs^TXL^-exo group than in the MSCs-exo and PBS group (*p* < 0.05–0.001) (Fig. 6F). Similarly, in an in vitro experiment, after coculture with MSCs-exo or MSCs^TXL^-exo for 24 h, H9C2 cells exhibited significantly increased levels of *miR-146a-5p* (*p* < 0.05–0.0001) with a much higher level in MSCs^TXL^-exo than in MSCs-exo (*p* < 0.0001) (Fig. 6G). To validate the vital role of *miR-146a-5p* in the cardioprotective effects of MSCs^TXL^-exo, *miR-146a-5p* loss-of-function studies were performed in MSCs^TXL^. MSCs^TXL^ were transfected with *miR-146a-5p* inhibitors (MSCs^TXL-inhibitors^) or its negative control (MSCs^TXL-inhibitors-NC^), and exosomes were then isolated from the conditioned medium. The efficiency of *miR-146a-5p* knockdown in both MSCs^TXL^ and its derived exosomes was quantified (Fig. 6H-I). Flow cytometry analysis showed that *miR-146a-5p* inhibition significantly increased MSCs^TXL^-exo-medicated apoptosis than inhibitors-NC (34.44 ± 5.25% vs. 26.54 ± 4.80%, *p* < 0.05) (Fig. 6J, K, Additional file 1: Table S7). Western blot analysis also showed significantly increased expression of both pro-apoptotic Bax and cleaved-caspase 3 in H9C2 cells treated with MSCs^TXL-inhibitors^-exo compared to MSCs^TXL-inhibitors-NC^-exo (both *p* < 0.05) (Fig. 6L–N, Additional file 1: Table S7), indicating the vital role of *miR-146a-5p* in mediating the cardioprotective effects of TXL pretreatment.

Therefore, the above results demonstrated that TXL pretreatment enhanced the therapeutic efficacy of MSCs by upregulating exosomal *miR-146a-5p* levels.

### *miR-146a-5p* protected H9C2 cells from apoptosis by targeting IRAK1 and inhibiting the nuclear translocation of the NF-κB p65 subunit

**Fig. 7:**
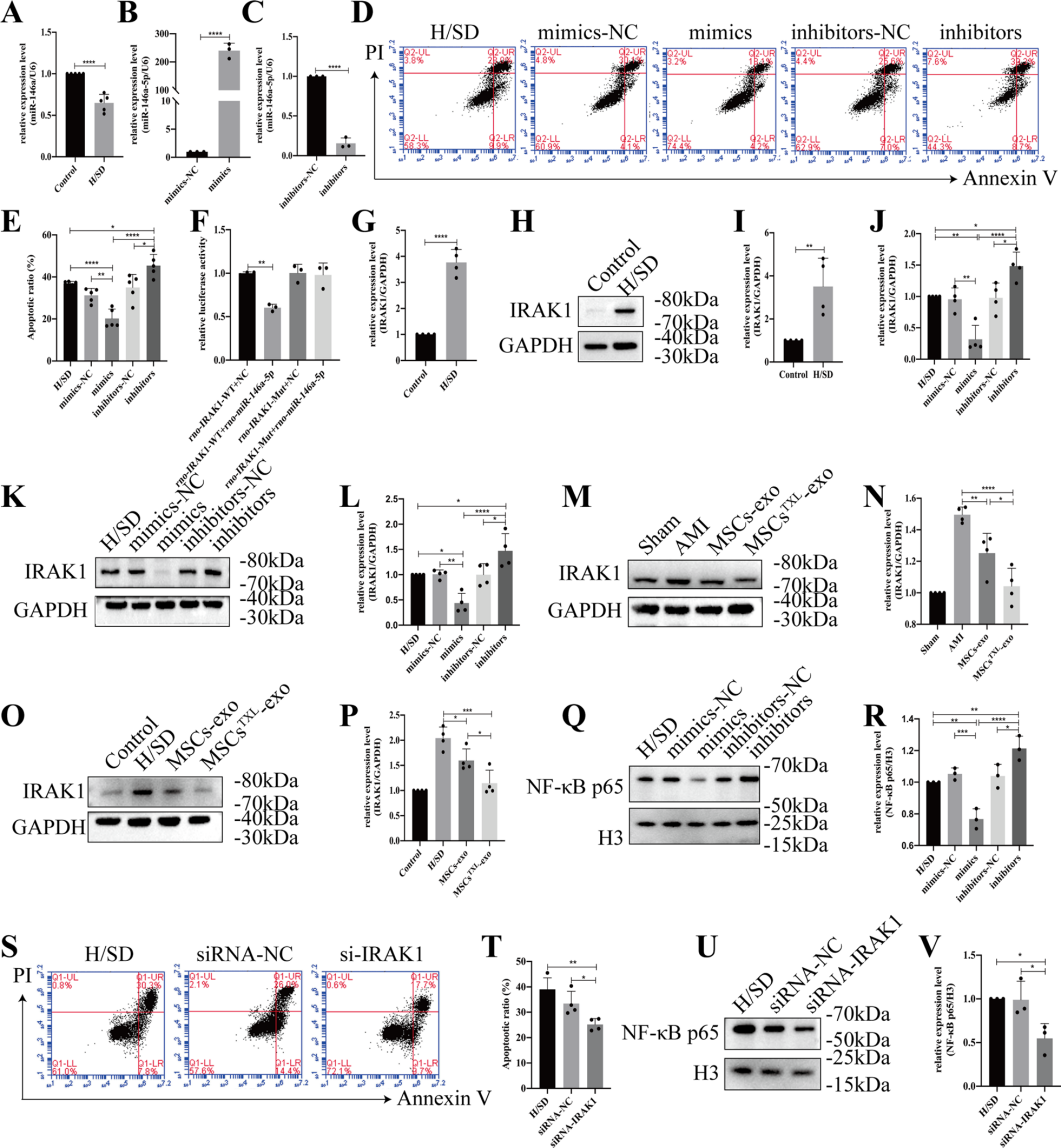
*miR-146a-5p* protected H9C2 cells from hypoxia injury by downregulating IRAK1 and inhibiting the nuclear translocation of the NF-κB p65 subunit. **A** The *miR-146a-5p* level of H9C2 cells was significantly reduced under H/SD conditions (*n* = 5). *miR-146a-5p* mimics significantly increased (**B**) or *miR-146a-5p* inhibitors significantly decreased (**C**) *miR-146a-5p* levels in H9C2 cells (*n* = 3). Representative images of the flow cytometry assay (**D**) and quantification (**E**) of apoptotic H9C2 cells transfected with *miR-146a-5p* mimics, inhibitors or the corresponding negative control (*n* = 5). **F** Luciferase activity assay of *miR-146a-5p* mimics-treated HEK293T cells, which overexpressed either IRAK1-wild-type or IRAK1-mutant. Under H/SD conditions, both the mRNA (**G**, *n* = 4) and protein levels of IRAK1 (**H-I**, *n* = 4) were obviously reduced. **J** The mRNA level of *Irak1* in H9C2 cells transfected with *miR-146a-5p* mimics, inhibitors or NC (*n* = 4). Representative western blotting images (**K**) and quantification (**L**) of the relative level of IRAK1 (*n* = 4). Western blotting images (**M** or **O**) and quantification (**N** or **P**) of the relative level of IRAK1 in MSCs^TXL^ treatment in vivo or in vitro. Representative images (**Q**) and quantification (**R**) of the relative level of NF-κB p65 (*n* = 3). Representative images of flow cytometry (**S**) and quantification (**T**) of apoptotic H9C2 cells transfected with IRAK1 siRNA or its NC (*n* = 4). Representative western blotting images (**U**) and quantification (**V**) of nuclear NF-κB p65 levels in H9C2 cells transfected with IRAK1 siRNA or its NC (*n* = 3). All data are presented as the mean ± SD. Statistical analysis was performed with one-way ANOVA or student’s t test. *p < 0.05, **p < 0.01, ***p < 0.001, ****p < 0.0001

The level of *miR-146a-5p* was examined in H9C2 cells after H/SD. The results showed that *miR-146a-5p* was significantly decreased under hypoxic injury (Fig. 7A), suggesting a possible connection between the reduction in *miR-146a-5p* and apoptosis of H9C2 cells under H/SD conditions. To further validate the protective role of *miR-146a-5p*, we directly tested the effects of *miR-146a-5p* mimics, inhibitors or respective NCs in H9C2 cells under H/SD conditions. *miR-146a-5p* mimics or inhibitors were confirmed to significantly increase or decrease *miR-146a-5p* levels in H9C2 cells, respectively (both *p* < 0.001) (Fig. 7B, C). Under H/SD condition, flow cytometry analysis demonstrated that *miR-146a-5p* mimics significantly reduced the cell apoptosis compared to mimics NC (20.22 ± 4.51% vs. 31.22 ± 3.35%, *p* < 0.01), whereas the inhibitors increased that compared to inhibitors-NC (45.44 ± 5.28% vs. 35.02 ± 6.22%, *p* < 0.05) (Fig. 7D, E), confirming the protective effects of *miR-146a-5p* on H9C2 cells under H/SD conditions.

Based on the prediction results of TargetScan*, Irak1* ranked highest among the predicted targets of *miR-146a-5p*, with two predicted target sites for *miR-146a-5p* in the 3′-untranslated region (3′-UTR) of the transcript of *Irak1* (Additional file 1: Table S6). To further confirm whether *miR-146a-5p* directly binds to the 3′-UTR of *Irak1*, we cloned the wild-type and mutant 3′-UTR of *Irak1* downstream of a firefly luciferase cassette in a luciferase reporter vector (Additional file 1: Fig. S3). As expected, *miR-146a-5p* mimics exclusively inhibited the luciferase activity of wild-type, suggesting that *miR-146a-5p* bound the *Irak1* mRNA 3′-UTR and thereby inhibited *Irak1* (Fig. 7F). Under H/SD conditions, both the mRNA and protein levels of IRAK1 in H9C2 cells were obviously increased (Fig. 7G-I). After transfection with *miR-146a-5p* mimics, inhibitors or the corresponding NCs, qPCR analysis indicated that *miR-146a-5p* mimics resulted in a significant decrease in *Irak1* mRNA levels (*p* < 0.01), while *miR-146a-5p* inhibitors resulted in a notably higher level (*p* < 0.05) (Fig. 7J). Western blot analysis further demonstrated that transfection with *miR-146a-5p* mimics resulted in a significant decrease in IRAK1 protein levels (*p* < 0.01) and inhibitors resulted in a significant increase in IRAK1 protein levels (*p* < 0.05) (Fig. 7K, L). More significantly decreased level of IRAK1 was detected in MSCs^TXL^-exo than MSCs-exo group both in vivo and in vitro (both *p* < 0.05) (Fig. 7M–P). We further detected the expression levels of NF-κB p65 and found that *miR-146a-5p* mimics suppressed NF-κB p65 subunit nuclear translocation (*p* < 0.001 vs. mimics NC), whereas transfection with *miR-146a-5p* inhibitors indicated the opposite result (*p* < 0.05 vs. inhibitors-NC) (Fig. 7Q, R). These results suggested the vital role of *miR-146a-5p* in inhibiting IRAK1 and NF-κB p65 subunit nuclear translocation in hypoxia-induced injury.

To further validate whether IRAK1 could regulate the nuclear translocation of NF-κB p65, we knocked down *Irak1* in H9C2 cells with siRNA. The knockdown efficiency of *Irak1* siRNA was validated by qPCR and western blotting (Additional file 1: Fig. S4). We found that knockdown of *Irak1* significantly reduced H9C2 cell apoptosis under H/SD conditions (*p* < 0.05 vs. siRNA-NC) (Fig. 7S, T) and further inhibited the nuclear translocation of NF-κB p65 in H9C2 cells in response to H/SD injury (*p* < 0.05 vs. siRNA-NC) (Fig. 7U, V).

Taken together, these results indicated that *miR-146a-5p* could protect H9C2 cells against hypoxic injury by inhibiting the IRAK1/NF-κB p65 pathway.

## Discussion

Post-AMI, massive cardiomyocyte loss and an intense inflammatory response result in deteriorated cardiac function and worse clinical outcomes [3–5]. Preventing the loss of cardiomyocytes early and suppressing inflammation in a timely manner are effective strategies in AMI treatment [43–45]. MSCs therapies have been recognized as promising for cardiac repair of AMI due to anti-apoptotic and anti-inflammatory effects [29, 46, 47], though the clinical efficacy of BMCs, including MSCs, was quite weak for AMI patients in clinical trials [8–11]. Therefore, numerous strategies have been developed to improve MSCs-based cardiac repair, including harsh microenvironment improvement in the infarcted region to promote the survival of implanted MSCs [19–21] as well as MSCs modifications [12–17, 26–28]. Although genetically modified approaches are the most effective strategy for increasing the therapeutic efficacy of MSCs, these approaches are currently infeasible in clinical practice and even suffer from difficulty achieving clinical translation. Instead, MSCs pretreated with drugs seem to be a promising solution to enhance cardiac repair effects with the potential for clinical translation in AMI treatment [26–28, 37].

TXL capsules, a Chinese medicine for patients with coronary artery disease [48–50], were reported in our previous studies to have cardioprotective effects through anti-apoptotic and anti-inflammatory mechanisms [24, 51–53], and to directly protect MSCs from apoptosis under H/SD conditions [23]. Therefore, we explored the effects of MSCs^TXL^ in enhancing cardiac repair in AMI and the main findings were as follows: (1) MSCs^TXL^ indeed facilitated cardiac repair with superior anti-apoptotic and anti-inflammatory effects at an early stage of AMI and achieved better cardiac function recovery via exosome secretion; (2) *miR-146a-5p* was enriched in MSCs^TXL^-exo (tenfold higher than that in MSCs-exo), which played a critical role in the marked cardioprotective effects of MSCs^TXL^-exo; (3) The transfer of exosomal *miR-146a-5p* into cardiomyocytes where IRAK1/NF-κB p65 signaling pathway was inhibited was at least partially mediated the above superior protective effects of MSCs^TXL^.

Inflammation in response to myocardium necrosis after AMI is the physiological process of cardiac repair [4, 5]; however, prolonged or excessive inflammation might lead to catastrophic consequences, such as further loss of cardiomyocytes and then impairment of systolic function, ventricular remodeling, matrix degradation or even cardiac rupture [44]. Thus, proper and timely inhibition of inflammation is the target of treatment for AMI [45, 54]. MSCs are promising for cardiac repair in the treatment of AMI due to their anti-inflammatory, anti-apoptotic and low immunogenicity properties [55]. Using a rat model of AMI, we first reported that intramyocardial injection of MSCs^TXL^ demonstrated better effects on cardiac repair than MSCs in reducing cardiomyocyte apoptosis and inflammation accompanied by better cardiac function recovery and smaller infarct size. In addition, the cardiac repair effects of MSCs^TXL^ were also dependent on secreted exosomes.

The cardioprotective effects of MSCs are primarily mediated by paracrine actions [29] and the transfer of exosome/EVs packed with bioactive proteins, RNAs and lipids [30, 31]. Modified MSCs, such as hypoxia- [13, 41] and GATA-4-modified MSCs [56], facilitated therapeutic effects mainly via secreted exosomes. Therefore, we confirmed in vitro that MSCs^TXL^-exo were superior to MSCs-exo in preventing H9C2 cells from undergoing apoptosis under H/SD conditions and further found that MSCs^TXL^-exo also had better cardiac repair effects than MSCs-exo in reducing apoptosis, suppressing inflammatory cytokines, increasing cardiac function and decreasing infarct size in vivo. Exosomes, in contrast to their parent cells, possess the distinct features of immune tolerability and product stability while retaining bioactivity, thus having the advantages of clinically repeatable administration and presenting a safer and more feasible option for clinical use [57]. Even xenogeneic exosomes were reported to recapitulate the entire benefit profile of auto- or allogeneic cardiosphere-derived cells (CDCs) without apparent side-effects in a preclinical study [58]. Considering that exosomes might be a promising cell-free and safe biological product in clinical use, MSCs^TXL^-exo would have great potential for clinical translation in AMI treatment.

Bioactive miRNAs packed in exosomes play key roles in the treatment of myocardial infarction or injury [36], by participating in inflammation modulation, cardiac repair and cardiac fibrosis [31, 59]. Thus, we performed exosomal miRNA sequencing and found the enriched *miR-146a-5p* to be the candidate effector of the outstanding cardioprotective effects of MSCs^TXL^-exo. Increased level of *miR-146a-5p* has been confirmed to decrease ischemia-related injury in different organs including the myocardium, cerebrum, intestine and kidney [60–63]. As a highly enriched miRNA in both CDC-exosomes and cardiac progenitor cell-exosomes, *miR-146a* is the critical agent mediating the cardioprotective effects of stem cell therapy [38, 40, 64]. The transfer of exosomal *miR-146a* has also been reported to inhibit apoptosis, promote the proliferation of cardiomyocytes and augment angiogenesis [38, 65]. We further found that *miR-146a-5p* was the highly enriched miRNA in MSCs^TXL^-exo (> tenfold higher than in MSCs-exo) and that MSCs^TXL^-exo yielded stronger than MSCs-exo in mediating cardioprotective effects in anti-apoptosis, anti-inflammation, cardiac function improvement and infarct size reduction. Since serum *miR-146a-5p* levels were significantly lower than those in healthy controls in the first 24-h-serum samples from ST-elevated MI (STEMI) patients [66], the early administration of MSC^TXL^-exo might be a promising strategy with clinical implications in STEMI patients.

After exploring the downstream mechanism of exosomal *miR-146a-5p*, we confirmed that it could downregulate the level of IRAK1. IRAK1 is a vital adaptor molecule in the Toll-like receptor (TLR) and IL-1 receptor (IL-1R) signaling cascades mediating the activation of NF-κB pathways [67–69]. *miR-146a-5p*-mediated downregulation of IRAK1 has been reported to suppress inflammatory responses in acute lung injury [70] and to decrease myocardial or renal ischemia/reperfusion injury [38, 60, 63]*.* Our data confirmed the vital role of IRAK1 downregulation in reducing cardiomyocyte apoptosis by IRAK1 knockdown. Previous studies have revealed that IRAK1 deletion disrupts the activation of NF-κB [71], which is associated with the inflammatory response and cell survival [72]. Our in vivo data found that downregulation of IRAK1 inhibited the nuclear translocation of NF-κB p65 and then protected cardiomyocytes against hypoxic injury. Therefore, our results demonstrated that exosomal *miR-146a-5p* upregulation by MSCs^TXL^ facilitated cardiac repair effects by inhibiting the IRAK1/NF-κB p65 signaling pathway.

Nevertheless, there are still several limitations in this study that should be noted. First, although intramyocardial injection is a highly effective route of MSCs or MSC-derived-exosomes delivery to the target area, other delivery routes, including intracoronary, especially intravenous injection, which are much more feasible in clinical use, deserve further study. Second, exosomes enriched in *miR-146a-5p* can also be taken up by other cell types in the heart which may exert other unknown effects. Third, other RNAs or proteins may exert synonymous or synergistic effects with *miR-146a-5p* which also remains to be studied. Finally, further preclinical studies including direct transplantation of MSCs^TXL-inhibitors^ and MSCs^TXL-inhibitors-NC^ to AMI models are needed to validate its efficacy and safety for clinical translation.

## Conclusions

Based on the anti-apoptotic and anti-inflammatory effects of the Chinese medicine TXL, our study highlighted the superior effects of MSCs^TXL^ in facilitating cardiac repair in AMI treatment and found that MSCs^TXL^ markedly enhanced cardiac repair via the transfer of exosomal *miR-146a-5p* to cardiomyocytes, which mediated cardioprotective effects by inhibiting the IRAK1/NF-κB signaling pathway. The study found a feasible strategy to enhance the therapeutic efficacy of MSCs and provided novel insights into the underlying mechanisms, which has clinical translational potential for AMI treatment.

## Supplementary Information

**Additional** **file 1.** Supplementary tables and figures.

## Data Availability

The datasets generated and analyzed during the current study are available in Gene Expression Omnibus (GSE184067), https://www.ncbi.nlm.nih.gov/geo/query/acc.cgi?acc=GSE184067.

## Abbreviations

BMCs: Bone marrow cells
MSCs: Mesenchymal stem cells
SD: Sprague–Dawley
TXL: Tongxinluo
AMI: Acute myocardial infarction
IRAK1: Interleukin-1 receptor-associated kinase
NF-κB: Nuclear factor-κB
miRNA: MicroRNA
H/SD: Hypoxia and serum deprivation
PBS: Phosphate-buffered saline
LAD: Left anterior descending coronary artery
TEM: Transmission electron microscope
NTA: Nanoparticle tracking analysis
LVEF: Left ventricular ejection fraction
LVFS: Left ventricular fractional shortening
LVEDV: Left ventricular end-diastolic volume
LVESV: Left ventricular end-systolic volume
LV: Left ventricle
HE: Hemoxylin-eosin
TUNEL: Terminal deoxynucleotidyl transferase-mediated dUTP nick-end labeling
NGS: Next-generation sequencing
NC: Negative control
qRT-PCR: Quantitative reverse transcription-polymerase chain reaction
TLR: Toll-like receptor
IL-1R: IL-1 receptor
CDCs: Cardiosphere-derived cells
