# Supplementary material for: Tongxinluo-pretreated mesenchymal stem cells facilitate cardiac repair via exosomal transfer of miR-146a-5p targeting IRAK1/NF-κB p65 pathway
Source: Stem Cell Res Ther. 2022 Jul 7;13:289. doi: 10.1186/s13287-022-02969-y (PMC9264662; doi:10.1186/s13287-022-02969-y)
Supplement: Supplementary file 1 — Additional file 1. Supplementary tables and figures. [file 13287_2022_2969_MOESM1_ESM.docx]

**Supplementary materials**

**Methods**

**Preparation of TXL Solution and TXL pretreatment**

TXL ultrafine powder (Shijiazhuang Yiling Pharmaceutical Co., Shijiazhuang, China) was dissolved in serum-free Iscove’s Modified Dulbecco’s Medium (IMDM, Gibco, USA). Then the suspension was sonicated for 30 minutes and centrifuged at 5000 rpm for 10 minutes. Sterile TXL solution was obtained by filtering the supernatant through a 0.22-μm filter. The precipitate was then dried, enabling precise weighing of the dissolved TXL powder. The solution was then adjusted to a final concentration of 2 mg/mL by adding IMDM and was then stored at 4 ^o^C or -20 ^o^C until use. The active constituents of TXL include paeoniflorin, ginsenoside Rg1, ginsenoside Rb1, jujuboside A, jujuboside B, isoborneol, and borneol.

For TXL pretreatment, when passages 3-4 MSCs grew to approximately 60%, TXL was added to its medium for 24h.

**Cell viability Assay**

To assess cell viability, 4 x 10^3^ MSCs were seeded per well in a 96-well plate. MSCs viability was determined by WST tetrazolium salt (CCK-8, Dojindo) according to the manufacturer’s protocol. Briefly, CCK-8 regent (10μL) was added to each well and the plates were incubated at 37 ^o^C for 3h. Absorbances at 450nm were then measured with a microplate reader.

**Apoptosis assay**

H9C2 cells were cultured in DMEM containing 10% FBS (Gibco) and penicillin (100U/mL)/ streptomycin (100 μg/mL) at 37°C with 5% CO_2_. After being plated on 6-well plates (1×10^5^ cells/well) and pretreated by exosomes for 24 hours or transfected with miRNA mimics, inhibitors or siRNA, H9C2 cells were then washed twice by PBS. The medium was changed to DMEM without glucose and FBS. Then H9C2 cells were put into a sealed GENbox hypoxic chamber fitted with an AnaeroPack (Mitsubishi Gas Chemical Company) to scavenge the free oxygen at 37 °C for 6 h.

**Immunofluorescence Staining**

For immunofluorescence staining, the paraffin sections were incubated with the antibodies at 4 ^o^C overnight, followed by goat anti-mouse (Thermo Fisher Scientific, A-11001, 1:200 dilution) or goat anti-rabbit (Thermo Fisher Scientific, A-11034, 1:200 dilution) highly cross-adsorbed Alex Fluro 488 or 594 secondary antibodies for 1 hour at room temperature. After washing, the nuclei were stained with DAPI. The sections were then observed under a laser scanning confocal microscope (Leica) at x400 magnification and five randomly chosen high-power fields (HPF) were measured per animal. The results of angiogenesis, reflected by α-smooth muscle actin (α-SMA, Abcam, 1:200 dilution), CD31 (Abcam, 1:300 dilution) staining were described as arteriole density and vascular density, respectively. Arteriole density was quantified as the number of α-SMA+ cells per HPF and vascular density was quantified as the number of CD31+ cells per HPF.

**Terminal Deoxynucleotidyl Transferase-mediated dUTP nick-end labelling (TUNEL) Assay**

To assess the apoptotic cardiomyocytes in the infarcted heart, *In Situ* Cell Death Detection kit (Roche) was used according to the manufacturer’s direction. The sections were blocked and incubated with TUNEL reaction mixture for 1 hour at room temperature, and then stained with cardiac Troponin T (cTNT, 1:200, Abcam) at 4°C overnight and nuclei were stained DAPI. The stained sections were examined under confocal microscope in four randomly chosen fields. Normal nuclei were presented as blue color, whereas apoptotic nuclei were green. All results were described as the percentage of apoptotic cardiomyocytes/total cardiomyocytes.

**Enzyme linked immunosorbent assay (ELISA) analyses**

Rat tumor necrosis factor (TNF)-α and rat interleukin (IL)-6 ELISA kits (eBioscience, USA, BMS625 and BMS622) were used to quantify the inflammatory cytokines TNF- α and IL-6 in the peri-infarcted myocardium. Heart tissue homogenates were prepared and the following procedures were performed according to the manufacturer’s instructions.

**Western blotting**

Cells, exosomes or tissues were collected and lysed in RIPA lysis buffer (Thermo Fisher Scientific) with a protease inhibitor cocktail (Roche), and protein concentrations were quantified by BCA protein assay (Beyotime). Proteins (20-60ug) were loaded with 4×loading buffer and separated on a 4-12% Bis-Tris gel (Invitrogen), transferred onto PVDF membranes (Millipore), blocked with 5% skim milk in TBST for 2 hours. Then, the membrane was incubated with specific primary antibodies against TSG101 (1:1000, Santa Cruz Biotechnology), CD63 (1:1000, Santa Cruz Biotechnology), Alix (1:1000, Cell Signaling Technology), cleaved-Caspase 3 (1:1000, Cell Signaling Technology), IRAK1 (1:1000, Invitrogen), NF-κB p65 (1:1000, Abcam), Histone H3 (1:2000, Cell Signaling Technology) and GAPDH (1:2000, Abcam) at 4°C overnight. Then, the membrane was incubated with the corresponding secondary antibodies (1:3000, Beyotime) for 1 hours at room temperature. Protein bands were detected by Chemiluminescence Imaging System (Tanno-5800multi). The densitometry of target bands was normalized to GAPDH or H3.

**Supplementary Tables**

**Supplementary table 1: List of specific primary antibodies**

| **Primary antibodies** | **Manufacturer** | **Catalog No.** | **Dilution** |
| --- | --- | --- | --- |
| CD90 Monoclonal antibody | Invitrogen | 45-0900-80 | 1:200 |
| CD29 Monoclonal antibody | Invitrogen | 12-0291-81 | 1:100 |
| CD45 Monoclonal antibody | Invitrogen | 11-0461-80 | 1:100 |
| CD11 Monoclonal antibody | Invitrogen | MA1-21592 | 1:100 |
| FITC Anti-CD31 antibody | Abcam | ab33858 | 1:200 |
| Anti-TSG101 antibody | Santa Cruz Biotechnology | sc-7964 | 1:1000 |
| Anti-CD63 antibody | Santa Cruz Biotechnology | sc-5275 | 1:1000 |
| Alix Mouse Monoclonal antibody | Cell Signaling Technology | 2171 | 1:1000 |
| CD73 Polyclonal antibody | Proteintech | 12231-1-AP | 1:1000 |
| Anti-CD31 antibody | Abcam | ab222783 | 1:200 |
| Anti-αSMA antibody | Abcam | ab124964 | 1:200 |
| Anti-Cardiac Troponin T antibody | Abcam | ab209813 | 1:200 |
| Anti-GAPDH antibody | Abcam | ab181602 | 1:3000 |
| Anti-IRAK1 antibody | Invitrogen | PA5-19855 | 1:1000 |
| Cleaved-Caspase 3 Rabbit Monoclonal antibody | Cell Signaling Technology | 9664 | 1:1000 |
| Bax antibody | Cell Signaling Technology | 2772 | 1:1000 |
| Histone H3 Rabbit Monoclonal antibody | Cell Signaling Technology | 4499 | 1:1000 |
| Anti-NF-κB p65 antibody | Abcam | ab16502 | 1:1000 |

**Supplementary table 2: Primers used for qRT-PCR**

| Gene name | Primer sequence (5’-3’) |
| --- | --- |
| *Gapdh* | Forward: 5’-GGCCAAGGTCATCCATGA-3’  Reverse: 5’-TCAGTGTAGCCCAGGATG-3’ |
| *Irak1* | Forward: 5’-CATCAAGCCAAGCCCAGAGA-3’  Reverse: 5’-GAAGTTGCAAGTGCCTTGGG-3’ |

**Supplementary table 3: Comparisons of cardiac function, infarct size, collagen area, angiogenesis, apoptosis and inflammation among the different AMI groups in rats.**

|  | Sham | AMI | MSCs | MSCs^TXL^ | MSCs^TXL+GW4869^ |
| --- | --- | --- | --- | --- | --- |
| LVEF (%) | 73.15±5.90 | 34.48±4.54 | 42.10±3.83 * | 49.69±6.02****^#^ | 38.65±3.58^$$$^ |
| LVFS (%) | 43.58±5.46 | 17.30±2.56 | 21.60±2.19 | 26.31±3.92*** | 19.60±1.99^$$^ |
| LVEDV (μL) | 247.6±66.90 | 425.7±54.23 | 370.5±79.88 | 354.2±104.4 | 397.0±119.3 |
| LVESV (μL) | 68.65±31.06 | 278.4±35.62 | 215.8±54.52 | 180.4±66.09** | 245.3±81.90 |
| Infarct size (%) | 4.36±3.98 | 66.29±4.96 | 56.82±5.41* | 46.39±8.06****^#^ | 61.75±3.80^$$$^ |
| Collagen area (%) | 5.51±2.30 | 44.52±6.73 | 30.46±6.30** | 28.87±7.72*** | 33.88±3.60^*^ |
| α-SMA | 1.64±0.46 | 4.04±0.33 | 7.04±1.55* | 9.56±1.87**** | 6.24±1.68^$$^ |
| CD31 | 2.47±1.03 | 4.80±1.64 | 9.88±2.21*** | 12.16±0.91**** | 7.44±0.86^$$$^ |
| Apoptotic cardiomyocytes ratio (%) | 0.096±0.22 | 6.17±0.72 | 4.48±0.45*** | 3.55±0.57****^#^ | 4.87±0.26**^$$^ |
| Relative expression level (Bax/GAPDH) | 1.00±0.00 | 6.48±1.38 | 2.49±0.78*** | 1.54±0.95**** | 2.89±1.02*** |
| Relative expression level (cleaved-Caspase3/GAPDH) | 1.00±0.00 | 5.04±0.65 | 3.13±0.36*** | 1.57±0.47****^##^ | 2.85±0.53****^$$^ |
| IL-6 (pg/mL) | 1230±228.7 | 3544±533.3 | 3078±308.9 | 2349±359.3***^#^ | 3275±584.7^$$^ |
| TNF-α (pg/mL) | 1065±367.2 | 3695±468.9 | 3048±646.1 | 2421±344.6*** | 3168±488.8 |

For cardiac function analysis, n = 8-10 for each group. For the analysis of infarct size, collagen area, n = 6; for quantification of α-SMA positively stained arterioles or CD31 positively stained capillaries at the border zone, n=5; for quantification of apoptotic cardiomyocytes ratio, n = 5 (4 random fields per animal); for the relative level of Bax and cleaved-Caspase 3, n=4; for quantification of IL-6 and TNF-α levels in infarct border zone tissue of rat hearts, n=6. All data are expressed as mean ± SD and was analyzed with one-way ANOVA followed by Tukey’s test. *, *p*<0.05, **, *p*<0.01, ***, *p*<0.001, ****, *p*<0.0001 compared with AMI group; ^#^, *p*<0.05, ^##^, *p*<0.01 compared with MSCs group; ^$^, *p*<0.05, ^$$^, *p*<0.01, ^$$$^, *p*<0.001 compared with MSCs^TXL^ group.

**Supplementary table 4 Apoptotic ratio and relative expression level of Bax and cleaved-caspase3 of H9C2 cells after MSCs-exo and MSCs^TXL^-exo treatment.**

|  | Control | H/SD | MSCs-exo | MSCs^TXL^-exo |
| --- | --- | --- | --- | --- |
| Apoptotic ratio (%) | 5.72±1.62 | 43.78±4.70 | 33.34±6.07** | 25.22±3.35****^#^ |
| Relative expression level (Bax/GAPDH) | 1.00±0.00 | 1.98±0.45 | 1.43±0.17* | 1.16±0.15** |
| Relative expression level (cleaved-caspase3/GAPDH) | 1.00±0.00 | 4.25±0.37 | 3.10±0.23*** | 2.57±0.25****^#^ |

For apoptotic ratio, n=5 for each group; for relative expression level of Bax and cleaved-Caspase 3, n=4. All data are expressed as mean ± SD and was analyzed with one-way ANOVA followed by Tukey’s test. *, *p*<0.05, **, *p*<0.01, ***, *p*<0.001, ****, *p*<0.0001 compared with H/SD group; ^#^, *p*<0.05, ^##^, *p*<0.01 compared with MSCs-exo group.

**Supplementary table 5: Comparisons of cardiac function, infarct size, collagen area, angiogenesis, apoptosis and inflammation among the different AMI groups in rats.**

|  | Sham | AMI |  | MSCs-exo | MSCs^TXL^-exo |
| --- | --- | --- | --- | --- | --- |
| LVEF (%) | 74.63±4.24 | 34.76±3.59 |  | 46.88±3.22**** | 53.39±5.69****^#^ |
| LVFS (%) | 44.42±3.73 | 17.43±2.02 |  | 24.59±2.00*** | 28.78±3.73**** |
| LVEDV (μL) | 193.3±46.09 | 415.9±64.54 |  | 404.8±61.95 | 379.0±105.8 |
| LVESV (μL) | 49.89±18.00 | 270.6±38.49 |  | 215.8±39.94 | 178.4±58.75** |
| Infarct size (%) | 5.01±3.74 | 67.36±7.49 |  | 50.49±7.35** | 39.05±7.91****^#^ |
| Collagen area (%) | 3.68±2.82 | 45.16±5.81 |  | 32.21±5.80** | 25.60±5.52**** |
| α-SMA | 1.96±0.67 | 4.24±0.48 |  | 8.92±1.57*** | 10.84±1.81**** |
| CD31 | 2.68±1.05 | 5.16±1.23 |  | 11.20±2.36*** | 12.75±1.93**** |
| Apoptotic cardiomyocytes ratio (%) | 0.12±0.26 | 5.74±0.75 |  | 4.20±0.47** | 2.91±0.45****^##^ |
| Relative expression level (Bax/GAPDH) | 1.00±0.00 | 6.60±0.70 |  | 5.03±0.96* | 3.33±0.56****^#^ |
| Relative expression level (cleaved-caspase3/GAPDH) | 1.00±0.00 | 3.41±0.37 |  | 1.85±0.16**** | 1.33±0.18****^#^ |
| IL-6 (pg/mL) | 1480±526 | 4411±385.6 |  | 2985±725.3** | 2027±561.7****^#^ |
| TNF-α (pg/mL) | 1342±317.7 | 3939±1175 |  | 2871±653.1 | 2381±401.7** |

For cardiac function analysis, n = 7-8 for each group. For the analysis of infarct size, collagen area, n = 6; for quantification of α-SMA positively stained arterioles or CD31 positively stained capillaries at the border zone, n=5; for quantification of apoptotic cardiomyocytes ratio, n = 5 (4 random fields per animal); for the relative level of Bax and cleaved-Caspase 3, n=4; for quantification of IL-6 and TNF-α levels in infarct border zone tissue of rat hearts, n=6. All data are expressed as mean ± SD and was analyzed with one-way ANOVA followed by Tukey’s test. *, *p*<0.05, **, *p*<0.01, ***, *p*<0.001, ****, *p*<0.0001 compared with AMI group; ^#^, *p*<0.05 compared with MSCs-exo group.

**Supplementary table 6 Predicted consequential pairing of miR-146a-5p and its two candidate target sites in the 3’UTR of IRAK1.**

|  | **Predicted consequential pairing of target region (top) and miRNA (bottom)** | **Site type** | **Context++ score** | **Context++ score percentile** | **Weighted context++ score** | **Conserved branch length** | **P_CT_** |
| --- | --- | --- | --- | --- | --- | --- | --- |
| Position 27-34 of IRAK1 3' UTR [rno-miR-146a-5p](http://www.mirbase.org/cgi-bin/mirna_entry.pl?acc=rno-miR-146a-5p) | 5'   ...AGACUCAGAUGUCAAAGUUCUCA...                        \|\|\|\|\|\|\|  3'       UUGGGUACCUUAAGUCAAGAGU | 8mer | -0.41 | 98 | -0.41 | 6.604 | N/A |
| Position 43-50 of IRAK1 3' UTR [rno-miR-146a-5p](http://www.mirbase.org/cgi-bin/mirna_entry.pl?acc=rno-miR-146a-5p) | 5' ...GUUCUCAUUGUUGGA----AGUUCUCA...                  \|\|\|\|    \|\|\|\|\|\|\|  3'         UUGGGUACCUUAAGUCAAGAGU | 8mer | -0.37 | 98 | -0.37 | 6.081 | N/A |

**Supplementary table 7 Apoptotic ratio and relative expression level of Bax and cleaved-caspase3 of H9C2 cells after MSCs^TXL-inhibitors^-exo and MSCs^TXL-inhibitors-NC^-exo treatment.**

|  | H/SD | MSCs^TXL-inhibitors^-exo | MSCs^TXL-inhibitors-NC^-exo |
| --- | --- | --- | --- |
| Apoptotic ratio (%) | 44.22±1.72 | 34.44±5.25** | 26.54±4.80****^#^ |
| Relative expression level (Bax/GAPDH) | 1.00±0.00 | 0.75±0.15* | 0.52±0.13***^#^ |
| Relative expression level (cleaved-Caspase3/GAPDH) | 1.00±0.00 | 0.75±0.10* | 0.49±0.18***^#^ |

For apoptotic ratio, n=5 for each group; for relative expression level of Bax and cleaved-Caspase 3, n=4. All data are expressed as mean ± SD and was analyzed with one-way ANOVA followed by Tukey’s test. *, *p*<0.05, **, *p*<0.01, ***, *p*<0.001, ****, *p*<0.0001 compared with H/SD group; ^#^, *p*<0.05, ^##^, *p*<0.01 compared with MSCs^TXL-inhibitors^-exo group.

**Supplementary Figures**

**Supplementary figure 1**

**
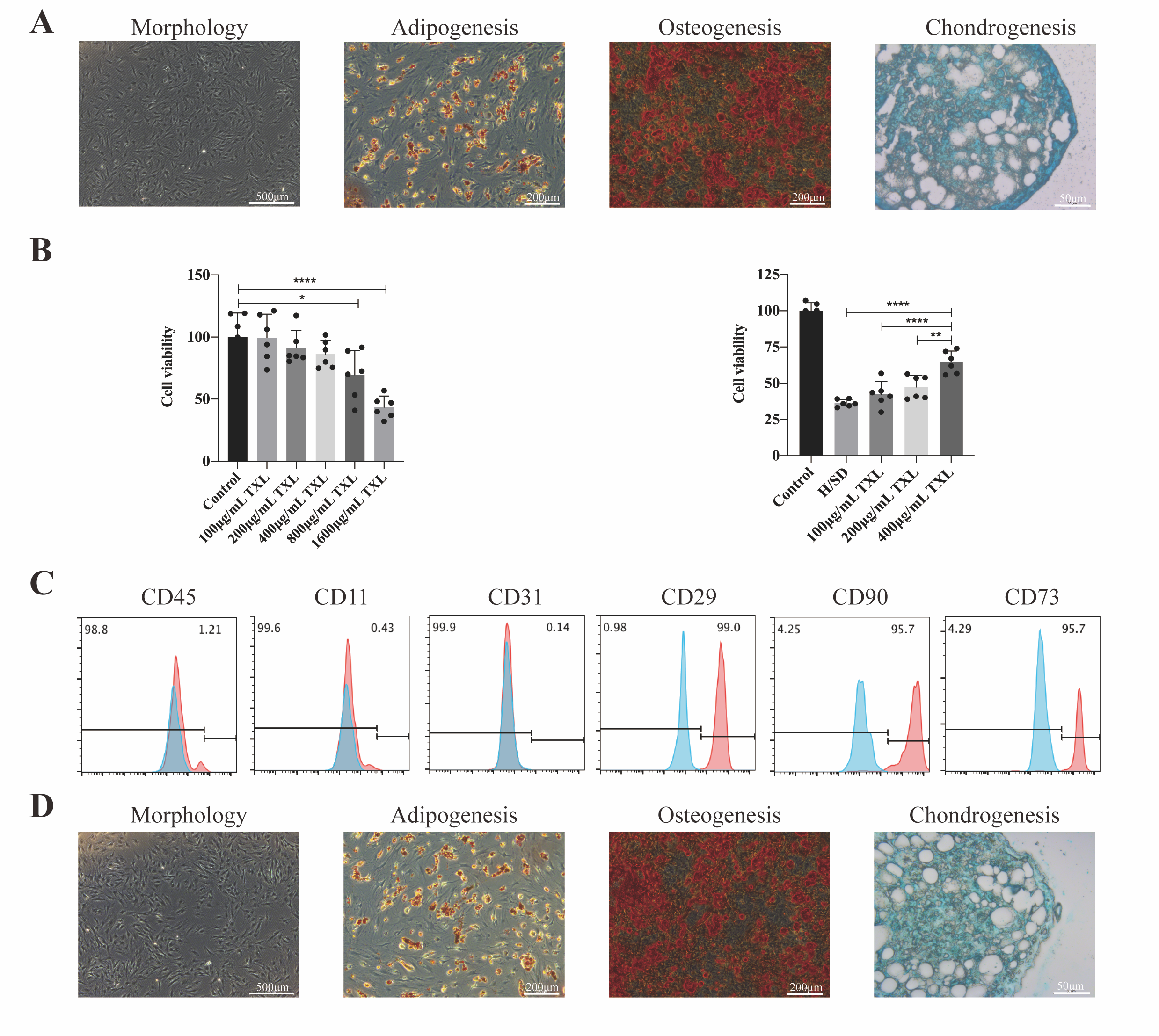
**

**Supplementary figure 1. Surface markers and differentiation ability of MSCs and MSCs^TXL^.** (A) Adipogenic differentiation, osteogenic differentiation and chondrogenic differentiation of MSCs. (B) Cell viability of MSCs pretreated with different concentration of TXL under normal or H/SD condition (n=6). Surface markers (C), adipogenic differentiation, osteogenic differentiation and chondrogenic differentiation (D) of MSCs^TXL^. All data are expressed as mean ± SD and was analyzed with one-way ANOVA followed by Tukey’s test. *, *p*<0.05, **, *p*<0.01, ***, *p*<0.001, ****, *p*<0.0001.

**Supplementary figure 2**

**
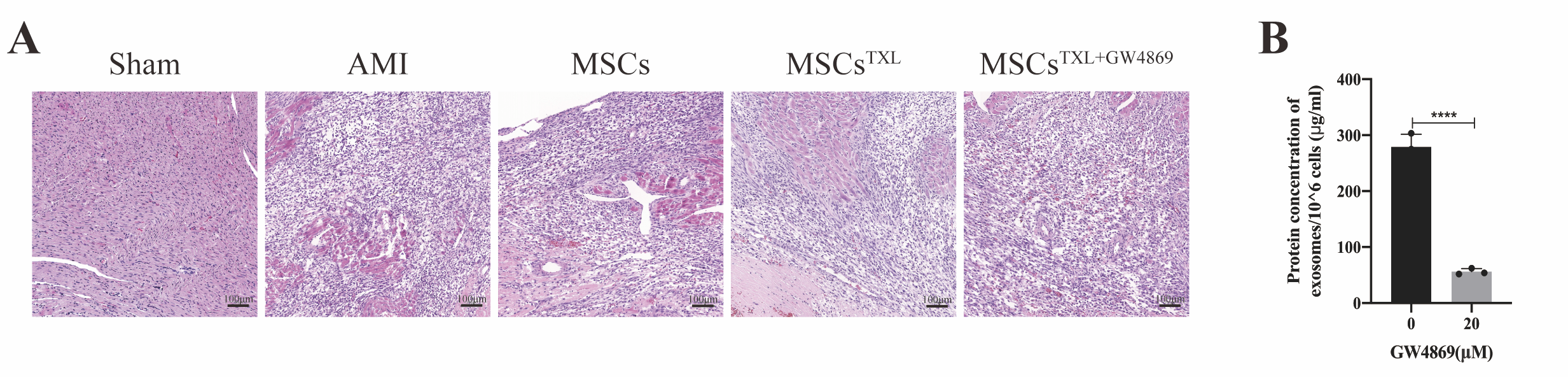
**

**Supplementary figure 2 Infiltration of inflammatory cells in the infarcted hearts and the effects of GW4869.** (A) Representative HE staining images at the border zone of the infarcted heart. Scale bar= 100 μm. (B) 20 μM GW4869 effectively blocked the release of exosomes (n=3).

**Supplementary figure 3**

**
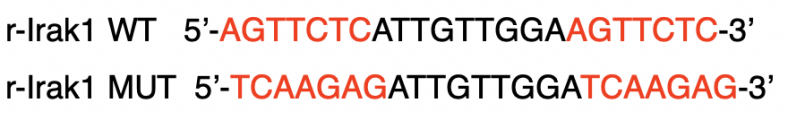
**

**Supplementary figure 3. The segment of IRAK1 3’UTR or a mutated segment.**

**Supplementary figure 4**


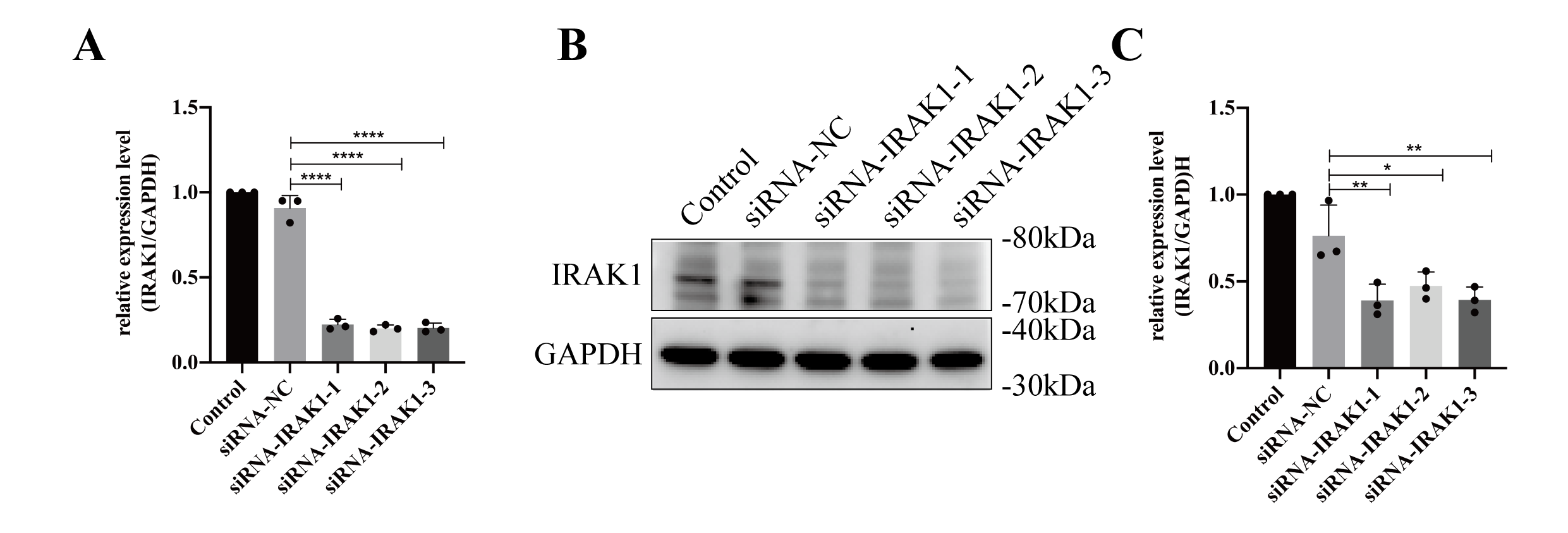


**Supplementary figure 4. The level of IRAK1 in H9C2 cells transfected with siRNA.** qPCR analysis (A), representative images of western blotting (B) and quantification of the protein level of IRAK1 (C) (n=3). siRNA-IRAK1-1 led to significant knockdown of the mRNA and the lowest protein level of IRAK1, so we used siRNA-IRAK1-1 in the following experiments.
